# Optical Coherence Tomography Angiography, Elastography, and Attenuation Imaging for Evaluation of Liver Regeneration

**DOI:** 10.3390/diagnostics15080977

**Published:** 2025-04-11

**Authors:** Svetlana Rodimova, Ekaterina Gubarkova, Nikolai Bobrov, Ilya Shchechkin, Vera Kozlova, Natalia Zolotova, Arseniy Potapov, Elena Kiseleva, Grigory Gelikonov, Natalia Gladkova, Vladimir Zagainov, Elena Zagaynova, Daria Kuznetsova

**Affiliations:** 1 Institute of Experimental Oncology and Biomedical Technologies, Privolzhsky Research Medical University, 10/1 Minin and Pozharsky sq., 603000 Nizhny Novgorod, Russia; kgybarkova@mail.ru (E.G.); daria.s.kuznetsova@gmail.com (D.K.); 2 The Volga District Medical Centre of Federal Medical and Biological Agency, 14 Ilinskaya St., 603000 Nizhny Novgorod, Russia; 3 Institute of Biology and Biomedicine, N.I. Lobachevsky Nizhny Novgorod National Research State University, 23 Gagarina Ave., 603022 Nizhny Novgorod, Russia; 4 A.V. Gaponov-Grekhov Institute of Applied Physics of the Russian Academy of Sciences, 46 Ulyanova Street, 603950 Nizhny Novgorod, Russia; 5 Nizhny Novgorod Regional Clinical Oncologic Dispensary, Delovaya St., 11/1, 603126 Nizhny Novgorod, Russia; 6 Lopukhin Federal Research and Clinical Center of Physical-Chemical Medicine of Federal Medical Biological Agency, 1a Malaya Pirogovskaya St., 119435 Moscow, Russia; 7 Laboratory of Omics and Regenerative Technologies, Institute for Regenerative Medicine, Sechenov First Moscow State Medical University (Sechenov University), 8-2 Trubetskaya St., 119991 Moscow, Russia

**Keywords:** liver regeneration, optical coherence tomography, OCT–elastography, OCT–angiography, attenuation coefficient of the OCT signal, image analysis

## Abstract

**Background/Objectives:** As a result of metabolic changes and the disruption of tissue architecture and microcirculation, the regenerative potential of the liver decreases with violations at both micro and macro levels. The development of intraoperative approaches for assessing its regenerative potential is important for reducing the risk of the occurrence of post-resection liver failure. In this study, we used multimodal optical coherence tomography (MM OCT), a combination of three optical coherence tomography modalities—OCT–angiography (OCTA), attenuation coefficient mapping, and OCT–elastography (OCE) to provide real-time three-dimensional and label-free assessment of changes in microcirculation, and in the structure and stiffness of the liver during regeneration. **Methods:** In our study, the regeneration of a healthy liver was induced by 70% partial hepatectomy. Monitoring of changes was carried out on the 0 (normal liver), 3rd and 7th day of regeneration using modalities of MM OCT. OCT offers the benefits of higher resolution and specificity compared with other clinical imaging modalities, and can be used, even intraoperatively. **Results:** By the 3rd day of liver regeneration, a decreased density of all observable vessels, together with increased values of the liver tissue’s attenuation coefficient and stiffness, was revealed compared to their initial state. However, by the 7th day, the studied parameters tended to return to their normal values, except that the density of large-caliber vessels continued to increase further. Histological and biochemical blood analysis methods were used to verify the MM OCT data. **Conclusions:** Such data are a first step towards further investigation of liver regeneration in pathology, and, taken in perspective, this should serve as a basis for predictive intraoperative assessment of the regenerative potential of the liver in a clinical setting.

## 1. Introduction

Curative surgery remains the gold standard treatment for liver tumors within a multimodal management approach [1]. Despite continuous improvements both in methods for preoperative assessment of liver condition and in surgical techniques (i.e., future liver remnant studies, portal vein embolization, staged hepatectomies, and venous liver deprivation), there still remain high risks of developing post-hepatectomic liver failure. Such failures are worrying, as are the severe complications that have a strong correlation with the extent of resection. The latter have around a 10% incidence after major liver resection, and their 90-day, post-surgery mortality rate can be as high as 60% [2]. This situation makes it especially necessary to determine the degree of regenerative potential of each individual patient’s liver to enable effective planning of the resection [1]. Liver failure after major resection mainly develops due to the presence of underlying hepatocellular carcinoma, hilar cholangiocarcinoma, or colorectal liver metastasis, which may leave patients with insufficient functional parenchyma following the operation [3,4]. An increased risk of developing liver failure is thus associated with the presence of underlying liver pathology or of individual variations in the liver’s regenerative potential [5].

Currently, there is no effective method for assessing the regenerative potential of the liver. Furthermore, clinicians also lack convenient methods for expressing the qualitative and quantitative assessment of the condition of the liver intraoperatively. After liver resection, several structural and vascular changes occur, including alterations in vessel density and diameter, the remodeling of the extracellular matrix, and the activation of hepatocyte and non-parenchymal cell proliferation. Disruption in any of these processes leads to a decrease in regenerative potential [6].

Previously, our research group identified key criteria for effective liver regeneration based on fluorescence bioimaging methods [7,8,9]. However, these methods do not provide information about the liver structure, nor about changes in blood circulation on a macro scale that are critical for assessing the regenerative potential of the liver.

Currently, optical bioimaging methods allow the intraoperative real-time study of various pathological processes at both cellular and tissue levels. OCT is a noninvasive imaging technique that provides real-time data on the functional and microstructural properties of tissues. It offers two- and three-dimensional images with higher resolution and specificity compared to other widely used clinical imaging modalities such as computed tomography, magnetic resonance imaging, and ultrasound. OCT also has advantages over histological analysis due to its noninvasiveness and the ability to analyze tissue architecture not only in one cross-sectional direction, but also in any plane thanks to its 3D imaging [10].

Extensions to OCT are starting to be widely used, such as the determination of the attenuation coefficient of the OCT signal [11,12,13], as well as polarization-sensitive (PS) OCT [14,15], OCT–angiography (OCTA) [16], and OCT–elastography (OCE) [17,18], which offer sensitive tools for the study of tissue microstructure, tissue microvasculature, and its elastic properties, respectively. These OCT modalities are being actively used, but, at present, mainly separately, for diagnostics with tumors of various localizations and for monitoring their therapeutic treatment both in experimental animals and in the clinic.

OCT has already proven its effectiveness for diagnosing liver diseases, in particular, in a model of non-alcoholic fatty liver disease in mice [19]. Here, when using 3D volumetric dynamic OCT, the researchers successfully visualized domains associated with inflammation and with lipid droplet accumulation. It has also been shown that a custom-built Jones-matrix-based PS OCT can be used for the label-free imaging of the functional and structural properties of the microvascular complex in mouse liver [20,21]. The use of MM OCT should therefore increase the available information on, and quality of, the predictive assessment of the volume of liver that must remain after resection to enhance the chances of successful recovery. However, studies devoted to the various physiological and pathological conditions of the liver using MM OCT are still few in number.

The aim of this work was to identify new optical criteria able to indicate effective liver regeneration, using MM OCT. In this study, for the first time, we combined in vivo functional characterization (by observing blood flow alterations using OCTA) with an ex vivo assessment of microstructural/morphological alterations (using the attenuation coefficient of the OCT signal and OCE) in liver tissue during regeneration.

## 2. Materials and Methods

### 2.1. Animal Model

**Figure 1:**
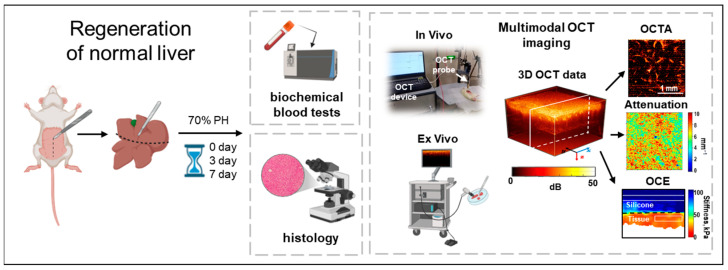
Road map of the experiment for the comprehensive study of normal liver regeneration using both standard verification methods and MM OCT.

The experimental study involved 14 male Wistar rats, each weighing between 250 and 350 g. To initiate the regeneration process, a 70% partial hepatectomy (PH) was performed on the animals [22]. Following the resection, each rat was kept in a clean cage under standard conditions in an SPF vivarium. We conducted monitoring of the liver tissue both in vivo and ex vivo, on the 3rd and 7th days after PH as well as prior to PH (day 0) (Figure 1).

Three biological groups were distinguished: 0th day (normal liver, *n* = 14), 3rd day (*n* = 7), and 7th day (*n* = 7). For each time point of the experiment, we obtained 2–3 fields of view for the liver tissue.

### 2.2. Evaluation of Liver Weight Recovery

To assess liver recovery, the organ’s weight was determined both before the induction of regeneration and then on the 3rd and 7th days of the recovery process. For the 70% PH, the initial liver weight was calculated according to the following formula: (weight of resected liver (g))/0.7. On the 3rd or 7th days, the weight of the regenerating liver was determined after euthanasia of the animal being tested.

### 2.3. Biochemical Blood Tests

Blood samples were collected from the tail vein of each rat. The samples were then centrifuged at 300× *g* for 15 min to separate the serum, and the concentrations of relevant materials in the serum samples were determined. Levels of aspartate aminotransferase (AST) and alanine aminotransferase (ALT), total protein (TP), urea, creatinine (Crea), and triglycerides (TGs) were recorded. The analysis was carried out using a semi-automatic biochemistry/coagulation analyzer (Dymind DP-C16, Shenzhen, China) and standard reagents (Diakon-DS, Moscow, Russia) in accordance with the manufacturer’s protocols.

### 2.4. Multimodal Optical Coherence Tomography (MM OCT) System and Data Acquisition

A spectral domain OCT system (IAP RAS, Nizhny Novgorod, Russia) was utilized, featuring a central wavelength of 1.3 µm, an axial resolution of approximately 15 µm, and a lateral resolution of around 20 µm in air, with an imaging speed of 20,000 A-scans per second [23,24]. The infrared laser power directed at the tissue was about 2 mW, with an acquisition time of 26 s. The optical probe was positioned in contact with the liver surface using an articulated arm. Three types of images—structural and angiographic in the en-face plane, and elastographic in the cross-sectional plane—were constructed in real time from the 3D data (4 × 4 × 1.5 mm) (Figure 1).

### 2.5. OCT Attenuation Coefficient Mapping

To analyze changes in the liver structure during its regeneration, the attenuation coefficient of the OCT signal was calculated. We used the depth-resolved method proposed by K.A. Vermeer [12] and modified by A. Moiseev [11]. The OCT signal attenuation coefficient distributions in the tissue in the en-face projection with averaging in the predefined depth range were constructed in the form of 2D color-coded maps. Such color coding enhances the tissue structure contrast compared to traditional OCT images by providing a more detailed visualization of the attenuation coefficient distribution [25]. For each collected A-scan set, attenuation coefficients were calculated in the predefined depth range starting from ~50 µm below the tissue surface to a depth of ~600 µm, to obtain representative images of the liver tissue morphology.

Quantitative values were calculated for the entire liver area and are presented as the mean ± standard deviation. Calculations were performed in an original program written in the Anaconda 4.3.1 mathematical environment (Python v. 3.6).

### 2.6. OCT-Based Angiography (OCTA) Imaging

Monitoring of the liver microvascular bed was performed in vivo using the OCTA modality. OCTA is based on an assessment of OCT signal speckle variations, which records a 3D picture of the tissue microcirculatory bed to the depth of OCT signal penetration. We used an algorithm to compensate for probe movements (due to breathing and heartbeat) [25]. The displacement compensation method based on phase difference compensation was used to process the OCT signal and ensure that artifact-free OCTA images were generated in real time. For OCTA analysis, each 3D image of the microvascular network was converted into a 2D en-face image using maximum intensity projection, allowing for the visualization of the vascular network across the entire depth of approximately 1 mm. It is essential to highlight that OCTA allows you to analyze perfused blood vessels. The OCTA images underwent post-processing—binarization and skeletonization [26]. The vessel density was calculated for both small- and large-caliber vessels, which was determined by the peculiarity of the liver blood supply. In the OCTA images, small-caliber vessels were defined as those with a diameter < 60 µm, while large-caliber vessels were those with a diameter ≥ 60 µm. Small-diameter vessels include interlobular veins (terminal portal venules), interlobular arteries (terminal hepatic arterioles), and central veins. These vessels participate in the blood supply of the hepatic lobule as a functional unit of the liver. Smaller sinusoids (hepatic capillaries) have a size of 5–8 μm, which is below the resolution of OCTA. Large vessels include branches of the portal vein, branches of the hepatic artery, and the collecting muscular venules [27]. Vessel density was calculated by multiplying the total number of skeletonized pixels by the area of a single pixel in square micrometers [28].

Density was calculated for the entire volume of the liver for which data were acquired, over the entire visualization depth of ~800 µm. Calculations were performed using a custom-written program in Python 3.6 (Anaconda 4.3.1).

### 2.7. OCT-Based Elastography (OCE) Imaging

The biomechanical properties (stiffness) of the liver tissue were studied in ex vivo samples using compression OCE based on the phase-sensitive visualization of the strains produced by the compression [18,29]. The axial interframe strain was estimated by finding the axial gradients of the interframe phase variations using the “vector” method proposed in [30] with additional improvements enabling a better quality of OCE maps in noisy conditions, as described in [31,32].

We used a reference layer of translucent silicone with pre-calibrated stiffness and a Young’s modulus of 40 kPa. This reference silicone layer was placed between the output window of the OCT probe and the surface of the tissue sample being studied. They were then slightly compressed using the OCT probe. The approach to OCT-image processing, developed in [33], enabled mapping of the compression-induced strains and quantitative estimation of the tangent elastic Young’s modulus (in kPa) for a preselected level of stress applied to the tissue. The tangent elastic Young’s modulus of the tissue is defined as the ratio of the incremental strain in the silicone multiplied by the silicone stiffness to the incremental tissue strain. In the resultant 2D OCE image, the resolution was about 4 times lower than that in the initial OCT images, i.e., ∼40–50 µm in both directions. The resultant OCE images were color-coded: the stiffer areas (lower strain) appear blue, while softer areas (higher deformation) appear red.

It should be noted that real tissues are usually pronouncedly nonlinear, so their Young’s modulus depends on the current stress. Therefore, for the quantitative estimates of Young’s modulus presented below, in all measurements, we used a “standardized” level of applied stress in the range from 1 kPa to 3 kPa centered at 2 kPa. For a comparative analysis of the stiffness (elastic Young’s modulus) using the values found for the liver tissue at the different time points, the ROI was selected to include the entire image (~500 × 3500 µm), excluding only the edges of the images and the upper border boundary with the silicone.

### 2.8. Histological and Morphometric Analysis

For the histological analysis, each liver sample was fixed in a 10% buffered formalin solution, dehydrated using isopropyl alcohol, and then embedded in paraffin. Sections that were 7 μm thick were deparaffinized and stained with hematoxylin and eosin following a standard protocol [34]. We captured 10–15 micrographs (at 400× magnification) for each sample using an EVOS M7000 microscope (Thermo Fisher Scientific Inc., Waltham, MA, USA). Morphometric analysis was conducted to evaluate hepatocyte proliferation activity. We measured the number of tetraploid hepatocytes, binucleate cells, and mitotic cells relative to 100 normal (non-dividing) cells [35].

### 2.9. Statistical Analysis

A statistical analysis of the morphometric analysis data, blood biochemical analysis, and data on attenuation coefficient, vessel density, and stiffness was calculated from 3D OCT, OCTA, and OCE images, respectively. The normality of the data distribution was assessed using the Shapiro–Wilk test. For the morphometric analysis data, the normality of data distribution was confirmed. For the biochemical blood test, using 3D OCT, OCTA, and OCE data, it was shown that normal distribution is not characteristic of all data sets. In this regard, the Mann–Whitney U-test was chosen to compare data sets. For each studied parameter, three time points were analyzed, and three paired comparisons were performed. To reduce the possibility of a type I error caused by multiple comparisons, the obtained *p*-value was multiplied by 3 (the number of comparisons), based on the Bonferroni multiple comparison correction method. In all cases, the differences were considered statistically significant when *p* < 0.05. The calculations were carried out using Prism 8.0.2 statistical software (GraphPad Software, San Diego, CA, USA). Morphometric and biochemical blood test data were presented using bar charts; outliers are not included. Box plots were used for the graphical presentation of the 3D OCT, OCTA, and OCE data.

## 3. Results

### 3.1. Weight Dynamics and Biochemical and Histological Evaluation of Liver Regeneration

The weight recovery percentage of the regenerating liver on day 3 was 80.9 ± 3.3%, and on day 7, it was 92.9 ± 1.1%.

**Figure 2:**
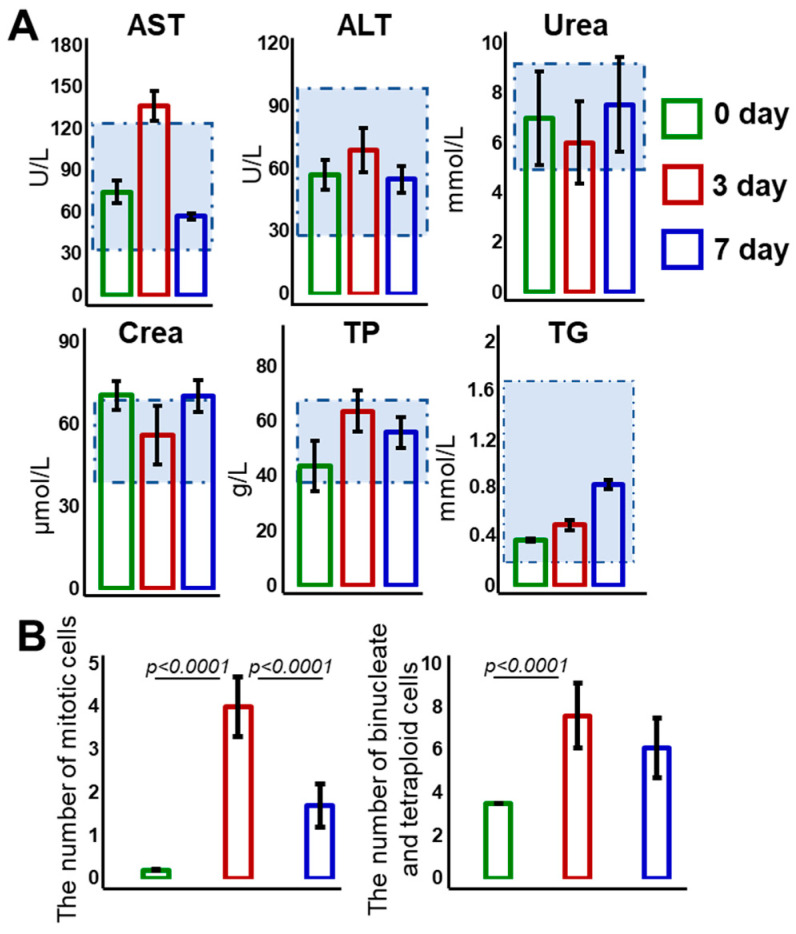
**Evaluation of liver function and hepatocyte proliferation activity in the liver before and during regeneration.** (**A**) Biochemical parameters in the blood serum of rats with induced liver regeneration. The area marked with a dotted line reflects the range of physiological values for each biochemical parameter under study. Mean ± standard deviation. (**B**) Morphometric analysis of the liver during regeneration. Values are presented as the number of dividing cells per 100 non-dividing cells. Mean ± standard deviation. Lines indicate a statistically significant difference between the studied groups, where *p* is the magnitude of the statistical significance of the differences between different groups.

All the biochemical parameters were generally within normal limits, with the exception of an increase in AST level seen on day 3, and a slight increase in urea level on day 7 of liver regeneration (Figure 2A). These findings confirm the absence of pathological abnormalities during regeneration and indicate preserved liver function.

**Figure 3:**
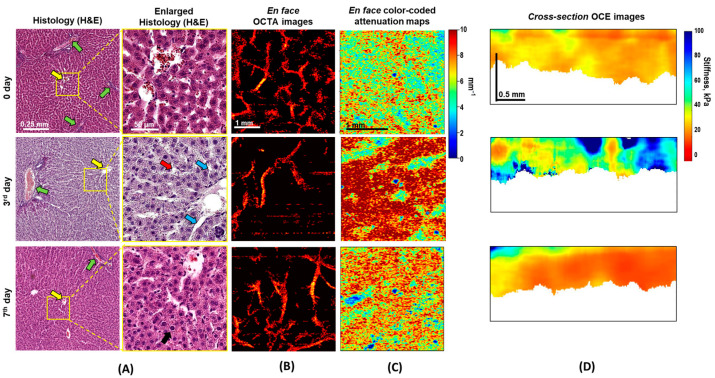
**MM OCT and histological examination of the liver during regeneration.** (**A**) En-face histological images of hematoxylin-and-eosin-stained liver tissue, where green arrows indicate interlobular veins; yellow arrows indicate central veins; the red arrow indicates large lipid droplets within hepatocytes; blue arrows point to irregularly dilated sinusoidal lumina; and the black arrow points to a large tetraploid hepatocyte. (**B**) En-face OCTA images show changes in vessel density (in vivo imaging). (**C**) En-face color-coded attenuation maps show the distribution of the attenuation coefficient. (**D**) Cross-section OCE images present the distribution of the stiffness values (ex vivo imaging).

On day 0, a histological analysis of the liver tissue (Figure 3A, top row) showed a typical lobular structure, without any architectural disturbances. There were no signs of inflammation (edema or cellular infiltrate). The levels of all studied biochemical blood parameters were within normal limits.

By day 3 of liver regeneration (Figure 3A, middle row), histological evaluation demonstrated structural changes in the liver parenchyma. Thus, the interlobular veins in the portal tracts were dilated and filled with blood; the central veins were also dilated, but no plethora was observed. The hepatocytes varied in size and were densely packed, due to reorganization (resorption) of the intercellular matrix. Infiltration with lipid droplets (microvesicular steatosis) was observed in the cytoplasm of the hepatocytes.

By day 7 of liver regeneration (Figure 3A, bottom row), the interlobular veins in the portal tracts remained filled with blood but were dilated to a lesser extent than on day 3. Lipid droplets could no longer be observed in the cytoplasm of the hepatocytes. Such transient steatosis is a characteristic change during the regeneration of a healthy liver [36]. The hepatocytes were enlarged, while their density had decreased, indicating restoration of the intercellular matrix. The remodeled lobules after liver restoration were large, had fuzzy boundaries, and contained unevenly dilated sinusoid networks.

A morphometric analysis showed a significant increase in the proliferative activity of the hepatocytes by day 3 of liver regeneration (Figure 2B).

### 3.2. Evaluation of Liver Microcirculation During Its Regeneration

**Figure 4:**
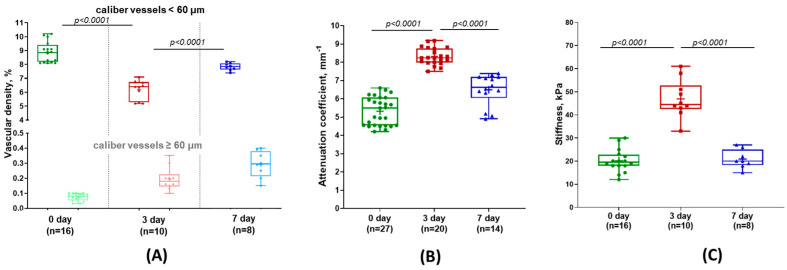
**Quantitative analysis of the vessel density, attenuation coefficient, and stiffness values for different time points of normal liver regeneration.** (**A**) Averaged vascular density results for small (<60 µm)- and large (≥60 µm)-caliber vessels, derived from OCTA images. (**B**) Distribution of the attenuation coefficient values derived from OCT images. (**C**) Distribution of the stiffness (Young’s modulus) of liver tissue, derived from OCE images. Center line in the boxplots—median; +—mean; box limits—25th and 75th percentiles; whiskers—minimum and maximum values. Lines indicate a statistically significant difference between the study time points (Mann–Whitney U-test with a Bonferroni correction for multiple comparisons), where *p* is the magnitude of the statistical significance of the differences between the different experimental time points.

Figure 3B shows representative OCTA images with the microvascular networks typical of normal liver before resection (day 0), and on the 3rd and 7th days after PH. The OCTA images on day 0 exhibit a uniform vascular network including blood vessels of different calibers (Figure 3B, top row). A quantitative analysis of the OCTA images showed that the density of small-caliber vessels (with diameters < 60 µm) in the normal liver was 8.8 ± 0.7% which was significantly higher than the density of the large-caliber vessels (with diameters ≥ 60 µm) (0.07 ± 0.02%) (Figure 4A, green and light green boxes).

On day 3 of liver regeneration, a marked depletion of the vascular network was observed. OCTA showed a decrease in the density of perfusing vessels, with irregular distributions of thick branched vessels (Figure 3B, middle row). The main contribution to the reduction in vessel density was made by a sharp decrease in the number of visualized small-caliber vessels (<60 μm), so large-caliber vessels (≥60 μm) began to predominate (Figure 4A, red and light red boxes). The density of remaining (undamaged) functional vessels with diameters < 60 µm after PH was 6.2 ± 0.6% (~1.5 times lower relative to day 0), while that of vessels with diameters ≥ 60 µm after PH was 0.2 ± 0.03% which was ~3.5 times greater than on day 0 (Figure 4A). An increase in vessel density ≥ 60 µm is explained by compensatory dilation of the branches of the portal vein, as a result of portal hypertension [37]; a decrease in vessel density < 60 µm is likely due to a sharp decrease in blood flow through the hepatic artery.

By day 7 of liver regeneration, the OCTA demonstrated the restoration of blood flow (Figure 3B, bottom row): irregularly arranged branched blood vessels were observed. The OCTA images demonstrated densely located clusters of irregularly arborizing large-caliber vessels. The vessel density was significantly higher than on day 3 after resection: the density of small-caliber vessels (<60 μm) rose to 7.8 ± 0.3% vs. 6.2 ± 0.6% on the 3rd day, *p* < 0.0001 (Figure 4A, blue and red boxes, respectively); for large-caliber vessels (≥60 µm), the growth was to 0.3 ± 0.08% vs. 0.2 ± 0.03% on the 3rd day (Figure 4A, light blue and light red boxes, respectively). This had mainly occurred as a result of an increase in the number of large-caliber vessels (Figure 3A, green arrow).

### 3.3. Attenuation Coefficient for Assessing Liver Tissue Structure

Figure 3C presents typical attenuation coefficient color-coded maps for normal liver on day 0 (top row), day 3 (middle row), and day 7 of regeneration (bottom row).

In the case of the normal liver, the attenuation coefficient maps (Figure 3C, top row) show a distribution of medium values of the attenuation coefficients in the liver tissue (4–8 mm^−1^) (Figure 3C, top row). On day 3, the attenuation coefficient maps show a relatively heterogeneous distribution of high (≥8 mm^−1^) and low (≤4 mm^−1^) attenuation coefficients (Figure 3C, middle row) compared to day 0. The higher attenuation coefficient values are associated with the dense arrangement of hepatocytes and with the deposition of small fat droplets (Figure 3A). The heterogeneous distribution of values is due to the dilated central veins (yellow arrows in Figure 3A), which do not scatter the signal and are represented on the attenuation coefficient maps as slit-like structures, having low values (yellow arrows in Figure 3C). On day 7 of liver regeneration, we showed a decrease in the attenuation coefficient (a return to the initial values seen on day 0) (Figure 3C, bottom row).

Figure 4 presents diagrams of the mean values of the calculated attenuation coefficients within the liver tissue for the three study groups (at different stages of regeneration). On day 3 after resection, the liver tissue was characterized by a statistically significant increase in the mean values of the attenuation coefficients compared to the normal liver (8.2 ± 0.5 mm^−1^ vs. 5.4 ± 0.7 mm^−1^, *p* < 0.0001) (Figure 4B, red and green boxes). By day 7, the values of the attenuation coefficient had decreased considerably to 6.4 ± 0.9 mm^−1^ (Figure 4B, blue boxes) and did not differ statistically significantly from values before liver resection.

### 3.4. Evaluation of Changes in Tissue Stiffness During Liver Regeneration

Figure 3D shows the OCE images demonstrating the changes in liver tissue stiffness during regeneration. The OCE images of normal liver (Figure 3D, top row) demonstrate a relatively low stiffness value (less than 20 kPa). The OCE image of the liver on day 3 of regeneration is mainly characterized by a significantly higher stiffness (more than 40 kPa) compared to the normal liver (Figure 3D, middle and top rows, respectively). This agrees with the histologically revealed increase in the number of proliferating hepatocytes of different sizes, and the accumulation of small lipid droplets in the hepatocyte cytoplasm (Figure 3A, middle row). On day 7, the stiffness values were similar to those of the normal liver on day 0 (≤20 kPa) (Figure 3D, bottom row), corresponding with the restoration of liver volume and completion of the regeneration process. Histological analysis showed that the sizes of the hepatocytes had become uniform again, confirming the restoration and remodeling of the ECM (Figure 3A, bottom row).

The results of a comparative evaluation of the stiffness values demonstrate a statistically significant twofold increase in tissue stiffness by day 3 of regeneration compared to the normal liver (36.2 ± 10.7 kPa vs. 17.1 ± 5.1 kPa; *p* < 0.0001) (Figure 4C, red and green boxes, respectively). On day 7, we observed statistically significant decreases in tissue stiffness compared to day 3 (20.0 ± 6.2 kPa vs. 36.2 ± 10.7 kPa; *p* < 0.0001) (Figure 4C, blue and red boxes, respectively).

Interestingly, although liver regeneration is considered complete by day 7, the MM OCT data indicate that the structure and the vascular network of liver tissues are not fully restored at that time.

Despite this, the data obtained using MM OCT are consistent with the data from standard verification methods. In particular, we showed the connection between high values of the stiffness coefficient and high proliferative activity of hepatocytes.

## 4. Discussion

This work is devoted to the identification of new predictive criteria of liver regenerative potential using three modalities of MM OCT. The obtained MM OCT data can potentially be applied to patients with both single-stage resections and two-stage resections, for which monitoring can be performed at the intermediate stage of liver regeneration.

To the best of our knowledge, this study is the first to combine different OCT modalities, such as OCTA, OCE, and attenuation coefficient estimation, to study the regenerative potential of normal rat liver tissue. This work has allowed us to investigate changes occurring in the microcirculation, structure, and stiffness of normal liver parenchyma both before and at different times post-hepatectomy. OCT is considered a very promising tool for surgeons due to the advantages of this method, such as safety (a near-infrared light source is used), accuracy (its micrometer scale resolution of ~10–15 μm), label-free visualization, and the high speed of obtaining 2D or 3D images of the subsurface tissue structure in real time to a depth of 2 mm. Continuous improvements in the visualization speed, sensitivity, development of functional modalities, and the emergence of endoscopic and handheld scanning probes have also led to increased interest in OCT [38,39,40].

Our MM OCT studies of rat liver tissue are consistent with previous studies using spectral domain OCT to assess attenuation coefficients [41] or microvasculature [19,21] in human and mouse liver. It has previously been shown that, in rodent liver, the value of the attenuation coefficient is mainly dominated by the scattering coefficient rather than the absorption coefficient [41,42].

Changes in tissue structure indicate possible pathological alterations in the liver, including inflammation, excessive lipid infiltration, and collagen accumulation. Previous studies, including those by our group [9,10] and others [43,44,45,46], have shown that such pathological changes significantly reduce the regenerative potential of the liver. In the work of Zhou F. et al. [42], it was demonstrated that the values of attenuation coefficients show significant differences between normal liver tissue and cancerous liver tissue in patients. In addition, because higher tissue density causes greater scattering, the attenuation coefficient can be taken to represent the tissue density. Therefore, in this study, an increase or reduction in the mean attenuation coefficient of the liver reflects a corresponding increase or reduction in the tissue density caused by a change in the number of hepatocytes and the deposition of fat droplets in their cytoplasm.

As is known, the liver has a unique blood supply, receiving approximately 25% of the cardiac output [47,48]. The vascular density directly correlates with the liver function and its regenerative potential. It affects the supply of nutrients from the intestine, affects liver function (xenobiotic detoxification and biosynthetic processes), and affects the supply of various bioactive substances that trigger hepatocyte proliferation (growth factors, cytokines, miRNA, and SiRNA). After 70% PH, the volume of blood flow through the remaining one-third of the liver increases sharply. As a result, massive hemodynamic changes occur within a few seconds [6]: there is an increase in blood pressure on the vessel walls, followed within an hour by an increase in the blood flow velocity and a resultant increase in the volume of blood passing through the liver [49]. Using intravital fluorescence microscopy in combination with epi-illumination, Dold et al. showed that after 70% PH in rats, the sinusoid diameter increased from 6.4 to 7.1 μm, while the sinusoidal density decreased from 45 to 42 n/mm. This was not due to an impairment of hepatic microvascular perfusion but rather to the relative hypermetabolism of the remnant liver [50].

The results we obtained with OCTA are consistent with the data of other authors regarding the microcirculatory network of liver tissue. In the work of Mukherjee P. et al. [19], it was shown that volumetric dynamic OCT can be used to visualize dynamic vessel-like structures in the normal liver. Our OCA image study demonstrated that normal rat liver possesses a dense vascular network with regularly shaped blood vessels of uniform diameter.

In this study, OCTA monitoring of normal liver regeneration also indicated microcirculatory restructuring occurring in the early stages of recovery (by day 3). At this stage, we observed avascular or vessel-poor zones in the OCA images, caused by the exclusion of blood flow in vessels with a diameter of less than 60 μm, which is probably associated with the reaction of the hepatic arterial buffer response (allows constant hepatic blood flow) [51]. After 70% PH, there is a sharp increase in pressure in the portal venous system, as the remaining liver tissue must then accommodate a relatively unchanged portal blood inflow despite the significant reduction in organ volume. At the same time, blood flow through the hepatic artery is reduced in a compensatory manner [47,51]. Thus, the decrease in blood vessel density observed with OCT is likely due to a sharp decrease in blood flow through the hepatic artery, while the portal vein branches increase in diameter. By day 7, the vessel densities returned to normal due to neoangiogenesis and the formation of new sinusoids, leading to a decrease in portal pressure because of the increased liver volume.

Another important parameter that influences the effectiveness of liver regeneration is tissue stiffness. Primarily, tissue stiffness depends on the composition and organization of the ECM. During liver regeneration, the extracellular environment is constantly changing. It begins with ECM degradation within 1 h of the PH, followed by its remodeling by stellate cells. Such degradation and remodeling of the ECM is an important activator of the proliferation of liver cells [52]. In this study, using OCE data, we observed a significant increase in stiffness by day 3 of regeneration and a return to baseline values by day 7. These results obtained using OCE are consistent with the data of other researchers. A number of authors have shown that a very stiff collagen matrix promotes hepatocyte proliferation [53,54], while hepatocytes within a soft collagen matrix remain in a non-dividing state (G0 phase of the cell cycle) [55]. Other authors have shown that hepatocyte proliferation is enhanced by the activation of integrin–YAP signaling and the initiation of the synthesis of cell adhesion proteins, which in turn also increases tissue stiffness [56]. In our work, we confirmed the high proliferative activity of hepatocytes on day 3 of regeneration, with a sharp decrease by day 7. Thus, we also confirmed the connection between high values of the stiffness coefficient and high proliferative activity of hepatocytes. However, it is worth noting that studying tissue stiffness alone is insufficient due to the lack of specificity of this indicator. In particular, high tissue rigidity not only triggers the proliferation of hepatocytes, but it also initiates the differentiation of hepatic stellate cells into myofibroblasts. Such a transition is then a trigger for the pathogenesis of fibrosis. That is why, for use in clinical monitoring, additional determination of changes in the structure and microvascularity of the liver tissue is necessary. In our study, this was carried out using the calculation of the attenuation coefficient of the OCT signal and vascular density.

There were some limitations in our study, starting with our OCT system, the speed and scan size of which were restricted due to the time constraints imposed by working with fresh tissues. As OCT technology is rapidly evolving, new systems are continually being developed, offering higher speeds and resolutions and wider scanning fields. Utilizing such improved systems in future work would allow us to overcome some of these problems. However, despite these limitations, our study demonstrated promising results, indicating its potential for future clinical applications. These include the opportunity for a rapid intraoperative assessment of liver regenerative potential and for the identification of the type and stage of pathology, enabling more effective resection procedures and a reduced risk of postoperative liver failure both in one-stage and two-stage resections.

## 5. Conclusions

Using our MM OCT-based approach, we identified criteria that indicate the progress of effective regeneration. By the 3rd day of regeneration, the following should be evident: (1) a significant increase in the attenuation coefficient and stiffness values, these being morphologically associated with the active proliferation of hepatocytes (seen as a variation in the sizes of the hepatocytes) and the accumulation of lipid droplets within them; (2) a decrease in the density of perfusing vessels and an increase in the diameter of individual vessels, which is morphologically associated with the plethora of lobular vessels. Then, by the 7th day of regeneration, the following should be seen: (1) an increase in the density of vessels with calibers ≥ 60 µm, and (2) decreases in both the attenuation coefficient and stiffness values, these being associated with a decrease in the number of lipid droplets and a termination of hepatocyte proliferation.

Our results indicate the significant potential of such visualization approaches for studying the structure, stiffness, and blood flow of liver tissue using MM OCT that could serve as a basis for future intraoperative clinical studies. Next, we plan to continue investigating liver regeneration, but this time under pathological conditions to identify criteria for identifying reduced liver regenerative potential. Furthermore, the application of machine learning or artificial intelligence approaches will accelerate the necessary MM OCT data processing by integrating attenuation, stiffness, and microcirculation indicators, thereby facilitating the clinical translation of this method.

## Data Availability

Data are available upon request from the corresponding author.

## Abbreviations

The following abbreviations are used in this manuscript:

MM OCT	Multimodal Optical Coherence Tomography
OCT	Optical Coherence Tomography
OCTA	Optical Coherence Tomography Angiography
OCE	Optical Coherence Tomography Elastography
PH	Partial Hepatectomy
ECM	Extracellular Matrix
AST	Aspartate Aminotransferase
ALT	Alanine Aminotransferase
TP	Total Protein
TGs	Triglycerides
